# Covid-Spiracy: Old Wine in New Barrels?

**DOI:** 10.1007/978-3-030-65355-2_2

**Published:** 2021-03-20

**Authors:** Peter Achterberg

**Affiliations:** 1 Tilburg University, Tilburg, The Netherlands; 2 Tilburg University, Tilburg, The Netherlands; 3 Tilburg University, Tilburg, The Netherlands; 4 Tilburg University, Tilburg, The Netherlands; Department of Sociology, Tilburg School of Social and Behavioral Sciences, Tilburg, The Netherlands

## Abstract

How to make sense of the current COVID-19 crisis? While many people rely on official statements made by governments, scientific institutions, and experts for answering this question, others do not. Recently, the Dutch newspaper NRC Handelsblad reported on people who adhere to the conspirational theory that the current COVID-19 crisis is linked to the introduction of 5G technology. These people point, for example, to so-called 5G experiments in the province of Wuhan, China, where the current COVID-19 crisis started in 2019. The “covid-spiracy” theory suggests that behind the societal curtains, elites are trying to deal with the problem of overpopulation by means of introducing 5G and blaming COVID-19 for the negative side effects. On Facebook and Twitter, people are actively discussing these theories with increasing momentum. And, inspired by theories on the adverse effects of 5G, people have tried to destroy 5G technology and hinder the spread of this technology in the Netherlands.

How to make sense of the current COVID-19 crisis? While many people rely on official statements made by governments, scientific institutions, and experts for answering this question, others do not. Recently, the Dutch newspaper NRC Handelsblad^1^ reported on people who adhere to the conspirational theory that the current COVID-19 crisis is linked to the introduction of 5G technology. These people point, for example, to so-called 5G experiments in the province of Wuhan, China, where the current COVID-19 crisis started in 2019. The “covid-spiracy” theory suggests that behind the societal curtains, elites are trying to deal with the problem of overpopulation by means of introducing 5G and blaming COVID-19 for the negative side effects. On Facebook and Twitter, people are actively discussing these theories with increasing momentum. And, inspired by theories on the adverse effects of 5G, people have tried to destroy 5G technology and hinder the spread of this technology in the Netherlands.

Meanwhile, the mainstream media (MSM) have been directing their attention to this newly developing phenomenon—the Volkskrant, NRC Handelsblad, De Telegraaf, Nieuwsuur, RTL-nieuws, and the NOS have all been reporting on people who link the current COVID-19 crisis to 5G. In many of these MSM reports, people with affinity to conspiracy theories are portrayed alongside some contextual information—fact checking the information provided by the covid spiracists or explaining their behavior. In this chapter, I analyze public reactions on Twitter to those MSM reports. Below, I first provide some background on research on conspiracy theories and then show how people perceive the covid-spiracy theory reports in the mainstream media.

## Research on Conspiracy Theories

Conspiracy theories can come in many guises, and, to their fundamental core, they are a set of beliefs that behind the societal curtains evil, malevolent groups are indoctrinating individuals and/or governing societies (Aupers 2012). In the last decade, scholarly attention for conspiracy theories (Aupers 2012; Douglas et al. 2019; Letort 2017; Locke 2009), and public support for such theories (Oliver and Wood 2014; Stempel et al. 2007) has risen.

These recent efforts have pointed out how much certain publics of Western societies actually believe in conspiracy theories (e.g., Oliver and Wood have shown that roughly one-quarter of the USA has an affinity with such theories) and point to two major factors that underlie beliefs in conspiracy theories. The first is that people are trying to make sense of the world they live in (Butter 2014; Grenier 1992; Popper 1945). As processes of individualization, globalization, and secularization rob people of their feelings of security, it is proposed that those who feel insecure are culturally rationalizing (Campbell 2015) and are trying to find the meaning and purpose of the things that are happening (Aupers 2012; Harambam and Aupers 2017).

The second factor in explaining affinity with conspiracy theories is modern-day anti- institutionalism (Elchardus and De Keere 2013; Melley 2008). Because people no longer trust the institutional backbone of modern societies, politics, science, the media, and the judiciary system, they start relying on themselves for finding out the truth and explaining what is going on.

## The Debate on Twitter

In the wake of MSM reports on the covid-spiracy theory on Twitter, people have been speaking their minds about such reports. In the following section, I will discuss public reactions to six of those reports.^2^

Among those who do not adhere to covid-spiracy theories, reactions can be divided into two camps. The first camp ridicules those who believe in the conspiracy theories, for instance, by implying that these people’s IQs need to be tested^3^ and that they are mentally deranged.^4^ One person mockingly asks “whether it would be worthwhile investigating the linkage between pizza Hawaii and the Coronavirus.”^5^ The second camp of opponents of the covid-spiracy diverge in their reactions to the MSM reporting in that some argue that the MSM are doing a great job in reporting on and debunking of conspirational thinkers^6^ whilst others argue that the MSM have crossed the line in giving conspirational thinkers a platform.^7^

Conspirational thinkers’ reactions can be divided into three overarching themes. The first is their expressed concern about 5G technology and COVID-19. They argue against having a source of electromagnetic radiation in their proximity and draw parallels to the “proven” negative effects of electricity pylons,^8^ and they point to the danger that lies within the adaptation of 5G technology.^9^ Note, however, that the focus of these arguments concerns the danger of 5G technology. Secondly, the covid-spiracy theory supporters react strongly against the MSM reports that, in their view, are making a mockery of conspirational thinkers. They argue that the MSM are “demonizing the truth” and that those who are really looking for the truth are portrayed as “nutcases”^10^ and that those who merely ask questions are being attacked unjustly.^11^ In addition, they complain that MSM reports are rude and too generalizing and they state that they should be “falsely accusing their own mothers.”^12^ Hence, it is safe to conclude that, in their eyes, the MSM are doing a particularly poor job. Thirdly and related to the foregoing, they state that the MSM are “slaves of the elite,”^13^^,^^14^ fully in line with the “current dictatorship of leftist political correctness” and dependent on a “dictatorial state”^15^ or on Bill Gates.^16^ They argue that “in view of the growing opposition pushing the introduction of 5G could be called militant”^17^ and others argue that there is just too little militancy in our country^18^—suggesting that demolishing a 5G antenna should be seen as a promising start of rising up against the elites.

## Conclusion

From my short and anecdotal discussion of responses to MSM reporting about the association between COVID-19 and 5G, it becomes clear that there are a lot of constants in the societal debates, which also thrived before the COVID-19 crisis started (Aupers 2012; Melley 2008). The first is that the MSM’s attention to conspirational thinkers who link the current COVID-19 crisis to 5G does not cause these conspirational thinkers to trust the MSM more. If anything, the attention paid by the MSM seems to cause more distrust. The more attention they pay to it, as they are doing it now, the more critical people with strong affinities with conspiracy theories will become. This reflects recent scholarship that suggests that a “rational” and balanced approach to conspiracy theories can be compared to pouring water on a grease fire (Palmer 2018).

The second is the anti-institutional perspective, which not only seems to affect the covid-spiracy supporters’ reactions but also the reactions of those who do not adhere to the theory. On both sides of the isle, people are critical to MSM reports, albeit for different reasons. Those with more affinity with the covid-spiracy link their rather critical view about the media to larger anti-institutionalist worldviews and see the victimization and demonization of conspirational thinkers portrayed in the MSM reports as a consequence of the media’s servitude to a corrupt and dictatorial elite. This suggests that the same mechanisms that underlie affinity with other conspiracy theories are at work here too. People try to make sense of what is going on (Aupers 2012) and combine it with an almost inflammatory anti-institutionalism (Melley 2008). In this sense, this covid-spiracy theory may be nothing but a theory based on the same mechanisms. Like old wine in new barrels.

## Footnotes

1 https://www.nrc.nl/nieuws/2020/04/19/nana-denkt-dat-de-overheid-ons-uitdunt-met-5g-tijdens-corona-a3997217

2 Reactions to these six reports were included here: (1) Telegraaf: https://www.telegraaf.nl/nieuws/715118123/weer-incident-bij-mast-verzet-5-g-wordt-militant, (2) Telegraaf: https://www.telegraaf.nl/nieuws/1762273884/complotdenkers-zien-verband-tussen-5-g-masten-en-corona?utm_source=twitter&utm_medium=social&utm_campaign=seeding-telegraaf, (3) RTLNieuws: https://www.rtlnieuws.nl/editienl/laatste-videos-editienl/video/5118871/kritische-arts-gaat-viral?fbclid=IwAR0qwJHL1U7yNDdBD516H5bmtDLluO4L5StkckTi74KS8Q2WLYaKjBJFpM4, (4) NRC Handelsblad: https://www.nrc.nl/nieuws/2020/04/19/nana-denkt-dat-de-overheid-ons-uitdunt-met-5g-tijdens-corona-a3997217, (5) NOS: https://nos.nl/artikel/2330065-brand-bij-vier-zendmasten-heel-sterk-vermoeden-van-brandstichting.html, and (6) Nieuwsuur: https://www.youtube.com/watch?v=RMuMBGaHttM&feature=youtu.be

3 https://twitter.com/terpGer/status/1252271787578097664

4 https://twitter.com/GJPvWesten/status/1248623467580125185

5 https://twitter.com/joyce_devivre/status/1252151070127726592

6 https://twitter.com/Genverbrander10/status/1252007731323494400

7 https://twitter.com/knillis/status/1252259095320637442

8 https://twitter.com/Kaasschaaf351/status/1248688462166994944

9 https://twitter.com/esmeraldaed/status/1248990551980027906

10 https://twitter.com/paulrikmans/status/1259762911951683584

11 https://twitter.com/IvoValkenburg/status/1259910435228332034

12 https://twitter.com/VolkersBram/status/1259090705013669888

13 Ibid.

14 https://twitter.com/Protect_Nation/status/1260515202207973376

15 https://twitter.com/reiniertweet/status/1259166954213928960

16 https://twitter.com/Complot_Denker/status/1259910360980750337

17 https://twitter.com/Verzet_Exit/status/1248732344544100358

18 https://twitter.com/My_Name_Nobody_/status/1248842665115955201
